# Supplementary material for: The Differences in the Whole-Brain Functional Network between Cantonese-Mandarin Bilinguals and Mandarin Monolinguals
Source: Brain Sci. 2021 Mar 2;11(3):310. doi: 10.3390/brainsci11030310 (PMC8000089; doi:10.3390/brainsci11030310)
Supplement: Supplementary file 1 [file brainsci-11-00310-s001.pdf]

The differences in the whole-brain functional network between Cantonese-Mandarin bilinguals and Mandarin monolinguals

*Xiaoxuan Fan[1], Yujia Wu[1], Lei Cai[1], Jingwen Ma[1], Ning Pan[1], Xiaoyu Xu[1], Tao Sun[1], Jin Jing[1], Xiuhong Li[1] **

Appendix

Methods and Materials

Table S1. Regions of interest (ROIs) in the Anatomical Automatic Labeling (AAL) atlas

Index	Regions	Abbreviations	Index	Regions	Abbreviations
1, 2	Precental gyrus	PreCG	47, 48	Lingual gyrus	LING
3, 4	Superior frontal gyrus, dorsolateral	SFGdor	49, 50	Superior occipital gyrus	SOG
5, 6	Superior frontal gyrus, orbital part	ORBsup	51, 52	Middle occipital gyrus	MOG
7, 8	Middle frontal gyrus	MFG	53, 54	Inferior occipital gyrus	IOG
9, 10	Middle frontal gyrus, orbital part	ORBmid	55, 56	Fusiform gyrus	FFG
11, 12	Inferior frontal gyrus, opercular part	IFGoperc	57, 58	Postcentral gyrus	PoCG
13, 14	Inferior frontal gyrus, triangular part	IFGtriang	59, 60	Superior parietal gyrus	SPG
15, 16	Inferior frontal gyrus, orbital part	ORBinf	61, 62	Inferior parietal, but supramarginal and angular gyri	IPL
17, 18	Rolandic operculum	ROL	63, 64	Supramarginal gyrus	SMG
19, 20	Supplementary motor area	SMA	65, 66	Angular gyrus	ANG
21, 22	Olfactory cortex	OLF	67, 68	Precuneus	PCUN
23, 24	Superior frontal gyrus, medial	SFGmed	69, 70	Paracentral lobule	PCL
25, 26	Superior frontal gyrus, medial orbital	ORBsupmed	71, 72	Caudate nucleus	CAU
27, 28	Gyrus rectus	REC	73, 74	Lenticular nucleus, putamen	PUT
29, 30	Insula	INS	75, 76	Lenticular nucleus, pallidum	PAL
31, 32	Anterior cingulate and paracingulate gyri	ACG	77, 78	Thalamus	THA
33, 34	Median cingulate and paracingulate gyri	DCG	79, 80	Heschl gyrus	HES
35, 36	Posterior cingulate gyrus	PCG	81, 82	Superior temporal gyrus	STG

37, 38	Hippocampus	HIP	83, 84	Temporal pole: superior temporal gyrus	TPOsup
39, 40	Parahippocampal gyrus	PHG	85, 86	Middle temporal gyrus	MTG
41, 42	Amygdala	AMYG	87, 88	Temporal pole: middle temporal gyrus	TPOmid
43, 44	Calcarine fissure and surrounding cortex	CAL	89, 90	Inferior temporal gyrus	ITG
45, 46	Cuneus	CUN			

The index of odd and even numbers represents brain regions of left and right hemispheres [1].

Definition of Global Network Metrics

The detailed description of the network metrics can be found in Rubinov and Sporns [2]. Global network metrics of a given network *G* with *N* nodes and *M* edges were defined as follows:

Clustering coefficient:

The clustering coefficient (C_p) measures the extent of local interconnectivity or cliquishness of a graph [3]. Here s_i is the strength of node *i*, and w_{ij} is the weight between node *i* and *j*. C_p is the averaged clustering coefficient of all nodes in a network.

$$C_p = \frac{1}{Ns_i(k_i - 1)} \sum\nolimits_{i,j \epsilon G} \frac{(w_{ij} + w_{ih})}{2} a_{ij} a_{ih} a_{jh}$$

Characteristic path length:

The characteristic path length (L_p) measures the mean length between pairs of nodes and quantifies the ability of information transmission [3]. Here L_{ij} is the shortest path length between node *i* and *j*.

$$L_p = \frac{1}{1/(N(N-1)) \sum_{i=1}^{N} \sum_{j \neq 1}^{N} 1/L_{ij}}$$

Global efficiency:

The global efficiency (E_g) captures the extent of information propagation in the network and is the inverse of the averaged characteristic path length between pairs of nodes within a network [4].

$$E_g = 1/L_p$$

Local efficiency:

The local efficiency (E_{loc}) is the average of the local efficiencies across all nodes [4]. Here G_i is the subgroup composed of the nearest neighbors of node *i*.

$$E_{loc} = \frac{1}{N} \sum\nolimits_{i \epsilon G} E_g(G_i)$$

Small-worldness:

To examine the small-worldness of functional brain network, we first generated 100 random networks containing the same number of nodes, edges, degree and weight distribution with real networks and calculated the mean $C_p(C_p^{rand})$ and mean $L_p(L_p^{rand})$ of these random networks. Then we compared the C_p and L_p of the real brain networks with those of random networks by calculating the γ(normalized $C_p = C_p^{real}/C_p^{rand}$) and λ(normalized$L_p = L_p^{real}/L_p^{rand}$). The network had small-world property if $\sigma > 1 (\sigma = \gamma/\lambda)$ [5, 6].

Results

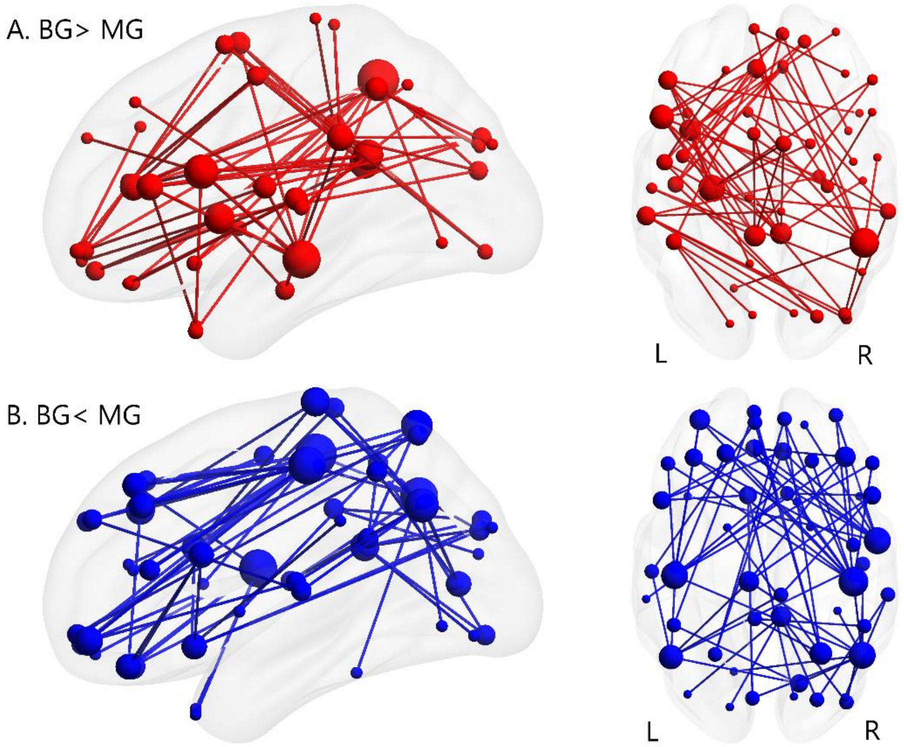

Fig S1. Whole-brain connectivity differences between bilinguals and monolinguals. Representation of the full bilinguals (BG) > monolinguals (MG) (red) and BG < MG (blue) networks showing significant group differences ($p < 0.0001$, corrected), before thresholding for visualization and discussion as shown in text Figure 1. Component sizes: BG > MG = 69 edges, BG < MG = 72 edges. The size of the spheres is determined by their number of connected edges in each subnetwork (i.e., larger nodes have more edges). Note that all figures are shown in neurological convention (subject-left is image-left): axial views are top-down.

A. Union mask

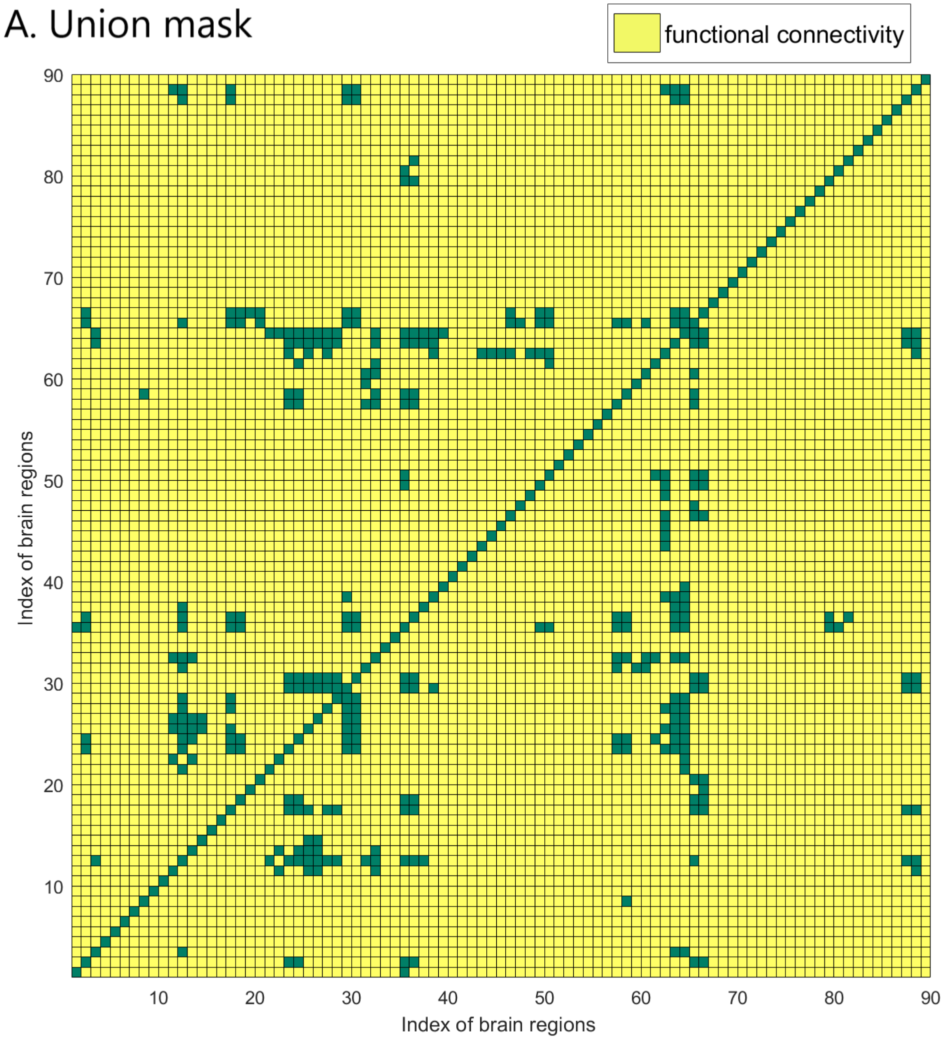

functional connectivity
90
80
70
60
50
40
30
20
10
Index of brain regions
10
20
30
40
50
60
70
80
90
Index of brain regions

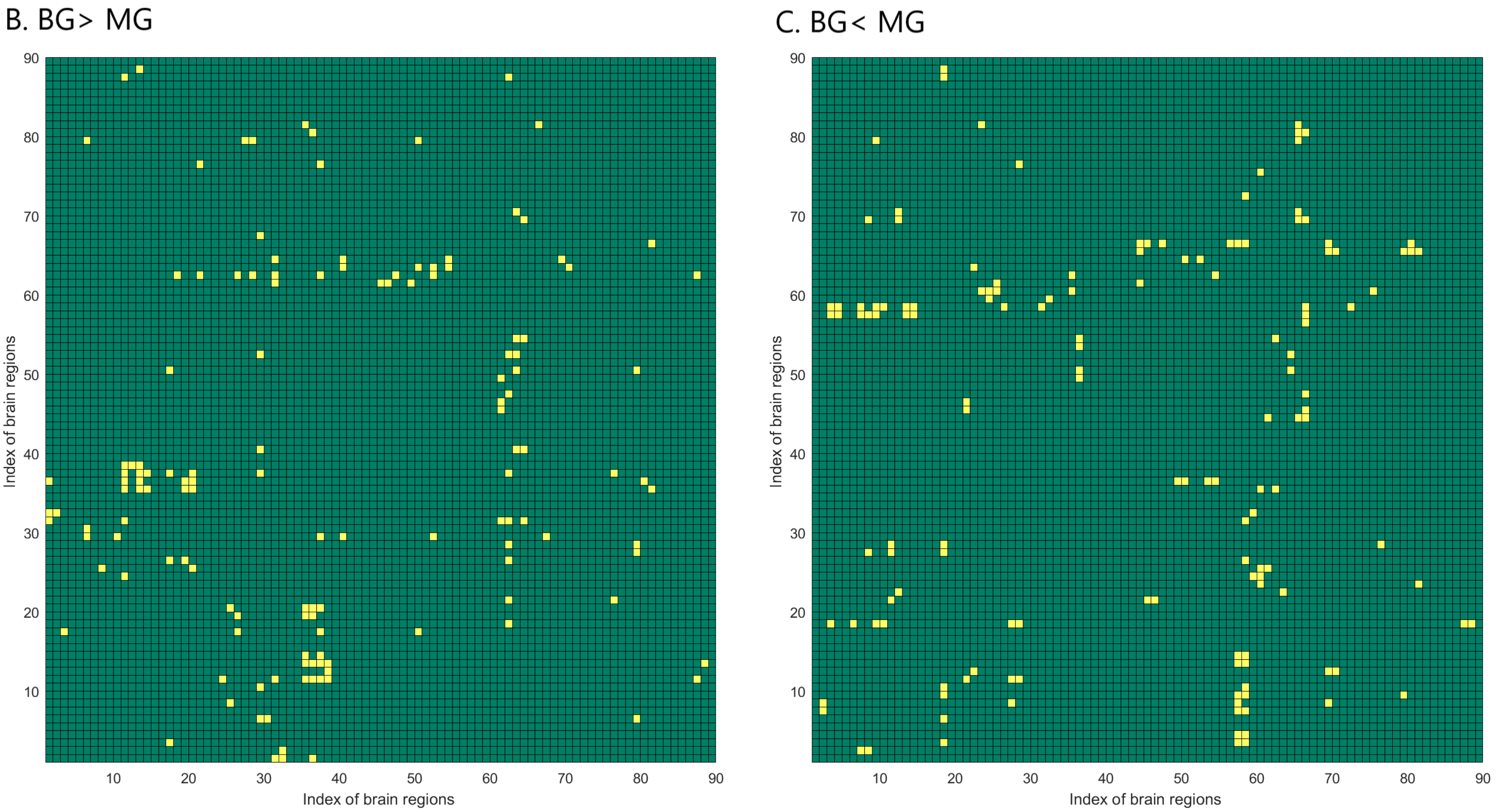

Fig S2. The Matrix of the union mask, and the Matrices of brain networks different between bilinguals and monolinguals (yellow squares represent functional connectivity, while green squares represent no functional connectivity). Figure A represents the union mask containing connectivity that was significant in either of the two groups. Figure B represents the brain network of bilinguals (BG) > monolinguals (MG) (69 edges, $p < 0.0001$, corrected), and Figure C represents the brain network of BG < MG (72 edges, $p < 0.0001$, corrected). Note that the index of brain regions is consistent with index in AAL atlas in Table S1.

Table S2. Selected node connectivity profiles. All coordinates given in MNI space

**Node A**	BG > MG connections			MG > BG connections		
	#	Region	Coordinate (x, y, z)	#	Region	Coordinate (x, y, z)
	1	ROL.R	(52.65, -6.25, 14.63)	1	PCG.L	(-4.85, -42.92, 24.67)
	2	OLF.L	(-8.06, 15.05, -11.46)	2	IOG.R	(38.16, -81,99, -7.61)
	3	ORBsupmed.R	(8.16, 51.67, -7.13)			
	4	REC.R	(8.35, 35.64,-18.04)			
	5	ACG.L	(-4.04, 35.40, 13.95)			
	6	HIP.L	(-25.03, -20.74, -10.13)			
	7	LING.L	(-14.62, -67.56, -4.63)			
	8	MOG.R	(37.39, -79.70, 19.42)			
	9	TPOmid.L	(-36.32, 14.59, -34.08)			
**Node B**	BG > MG connections			MG > BG connections		
	#	Region	Coordinate (x, y, z)	#	Region	Coordinate (x, y, z)
	--	--	--	1	SFGdor.L	(-18.45, 34.81, 42.20)
				2	SFGdor.R	(21.90, 31.12, 43.82)
				3	MFG.L	(-33.43, 32.73, 35.46)
				4	ORBmid.L	(-30.65, 50.43, -9.62)
				5	ORBmid.R	(33.18, 52.59, -10.73)
				6	IFGtriang.L	(-45.58, 29.91, 13.99)
				7	IFGtriang.R	(50.33, 30.16, 14.17)
				8	ORBsupmed.R	(8.16, 51.67, -7.13)
				9	ACG.L	(-4.04, 35.40, 13.95)
				10	ANG.R	(45.51, -59.98, 38.63)
				11	CAU.R	(14.84, 12.07, 9.42)
**Node C**	BG > MG connections			MG > BG connections		
	#	Region	Coordinate (x, y, z)	#	Region	Coordinate (x, y, z)
	1	PreCG.L	(-38.65, -5.68, 50.94)	1	SOG.L	(-16.54, -84.26, 28.17)
	2	IFGoperc.L	(-48.43, 12.73, 19.02)	2	SOG.R	(24.29, -80.85, 30.59)
	3	IFGtriang.L	(-45.58, 29.91, 13.99)	3	IOG.L	(-36.36, -78.29, -7.84)
	4	SMA.L	(-5.32, 4.85, 61.38)	4	IOG.R	(38.16, -81,99, -7.61)
	5	SMA.R	(8.62, 0.17, 61.85)			
	6	HES.R	(45.86, -17.15, 10.41)			
**Node D**	BG > MG connections			MG > BG connections		
	#	Region	Coordinate (x, y, z)	#	Region	Coordinate (x, y, z)
	1	SFGmed.R	(9.10, 50.84, 30.22)	1	OLF.L	(-8.06, 15.05, -11.46)
	2	ACG.L	(-4.04, 35.40, 13.95)	2	REC.L	(-5.08, 37.07, -18.14)
	3	PCG.L	(-4.85, -42.92, 24.67)	3	REC.R	(8.35, 35.64,-18.04)
	4	PCG.R	(7.44, -41.81, 21.87)			
	5	HIP.L	(-25.03, -20.74, -10.13)			
	6	HIP.R	(29.23, -19.78, -10.33)			
	7	TPOmid.L	(-36.32, 14.59, -34.08)			

ACG, anterior cingulate and paracingulate gyri; ANG, angular gyrus; BG, bilinguals; CAU, caudate

nucleus; HES, heschl gyrus; HIP, hippocampus; IFGoperc, inferior frontal gyrus, opercular part; IFGtriang, inferior frontal gyrus, triangular part; IOG, inferior occipital gyrus; L, left; LING, lingual gyrus; MFG, middle frontal gyrus; MG, monolinguals; MOG, middle occipital gyrus; OLF, olfactory cortex; ORBmid, middle frontal gyrus, orbital part; ORBsupmed, superior frontal gyrus, medial orbital; PCG, posterior cingulate gyrus; PreCG, precental gyrus; R, right; ROL, rolandic operculum; REC, gyrus rectus; SFGdor, superior frontal gyrus, dorsolateral; SFGmed, superior frontal gyrus, medial; SMA, supplementary motor area; SOG, superior occipital gyrus; TPOmid, temporal pole: middle temporal gyrus.

Correlation analysis

In order to explain the function network differences better, we did the correlation analysis between existing behavioral measures and functional network connectivity. Behavioral measures include visual and auditory phonological awareness, rapid naming, executive control and self-rated language proficiency. The behavioral measures and results are as follows:

1. Behavioral measures

1.1 Visual and auditory rhyming judgement tasks

The visual and auditory rhyming judgement tasks have been reported in our previous studies [7, 8]. For the visual rhyming judgement task, two paired words were displayed on the screen sequentially. Each word was presented for 800 ms with a 200-ms blank interval between words, which accounted for 1800 ms. After presentation of the words, a red fixation cross was displayed on the screen, indicating that the subjects should respond. Subjects were instructed to decide whether these pairs rhymed or not as accurately and quickly as possible.

For the auditory rhyming judgement task, two paired words were broadcasted in stereo sequentially. Each word lasted for 800 ms with a 200 ms blank interval between words, accounting for 1800 ms. Then, a red fixation cross was displayed on the screen, indicating that the subjects should respond. The response criteria were the same as those for the visual task.

1.2 Rapid naming

Four stimuli including colors, digits, letters and objects were used for this task. Stimuli were repeatedly presented visually in random order on a six row × five column grid. Participants were asked to name each digit in sequence as quickly as possible. Each participant completed the test twice, and the total time (s) taken to name all digits was collected, averaged and converted to a per-second score.

1.3 Behavioral tasks of executive function

1.3.1 Stroop Color and Word Test

The Stroop Color and Word Test was used in this study to evaluate the interference inhibition control ability [9]. As the main written language for the two groups was Mandarin, this test was conducted using Mandarin. In the task, the participants were presented with words written in different colors, and they were required to respond to the colors and ignore the meanings of the words. The experimental materials included four Chinese characters '红、黄、蓝、绿' and their corresponding colors 'red, yellow, blue, green'. Chinese characters with colors were presented one by one in the center of the computer screen, and the participants were required to press the corresponding keys ('red, yellow, blue and green' labels were attached to the keyboard) to respond to the colors of Chinese characters. There were three experimental conditions: consistent condition, the color of the word was consistent with the meaning of the word, for example, the color of '黄' is yellow; Conflict conditions, the

color of the word conflicted with the meaning of the word, for example, the color of ‘黄’ is red; Neutral condition, an ‘X’ with a color. Every ‘color-word conflict’ word appeared 6 times, with a total of 72 times; every "color-word consistent" word appeared 6 times, with a total of 24 times; every "X" appeared 6 times, with a total of 24 times. Three kinds of stimuli were presented one by one in random order. After introducing the experimental rules, the subjects can practice 10 times until they understood the rules. During the experiment, a fixation point appeared on the screen for 500ms, then stimulus materials appeared, and the next stimulus appeared after the subjects responded by pressing buttons. The interference effect in Stroop was calculated according to the formula: total time + ((2 × mean time per word) × number of response errors) [10].

1.3.2 GO/no-go

The go/no-go paradigm in this study was designed referring to previous study and was used to evaluate the response inhibition control ability [11]. The stimuli presented to the subjects were two letters F and J. If there was no red dot above the letter, it was a ‘go’ signal; and if there was a red dot above the letter, it was a ‘no-go’ signal. In the experiment, there was a fixation point for 500ms, and then the stimulus material was presented for 1000 ms. At this time, the subject made a judgment and pressed the button to respond, with an interval of 150ms between the end of the response and the presentation of the next stimulus. Participants were required to press F when ‘F’ appeared on the screen, J when ‘J’ appeared, and not press any key when there was a red dot above the letter. The results in go/no-go were calculated by the mean RT of correct response.

1.3.3 Shift

The shift was measured using color-shape switch paradigm [11]. The experimental materials included two objects (pentagram and circle) and two colors (red and blue), and there were four kinds of pictures (red pentagram, blue pentagram, red circle and blue circle). The first stage was to judge the color (or object) of the pictures, the second stage was to judge the object (or color), and the third stage was to judge according to the prompt words (color or object). Participants were required to press "F" when the color was blue and "J" when the color was red; press "F" when the object was circle and "J" when the object was pentagram. The results in color-shape switch were calculated by the mean RT of correct response.

2. Correlation analysis

First, we calculated the sum strength of functional connectivity within the ‘BG’ and ‘MG’ network for the bilinguals and monolinguals. Second, we did the correlation analysis between the brain network connectivity and behavioral measures for both bilinguals and monolinguals. Table S3 showed that the interference effect in Stroop Test was negatively correlated with the sum strength functional connectivity in the ‘BG’ network and ‘MG’ network for the bilinguals. It suggested that stronger brain network connectivity in the ‘BG’ and ’MG’ network was related to better interference inhibition control ability of bilinguals. Table S4 showed that the accuracy of auditory rhyming judgement task was negatively correlated with the sum strength functional connectivity in the ‘BG’ network and ‘MG’ network for the monolinguals.

Table S3. The correlation between the brain network connectivity and behavioral measures in bilinguals

	the sum strength of functional connectivity in 'BG' network	the sum strength of functional connectivity in 'MG' network

	r	*p*	*r*	*p*
Visual rhyming judgement task				
Accuracy of Mandarin	-0.186	0.352	-0.173	0.389
Response time of Mandarin	0.169	0.398	0.025	0.903
Accuracy of Cantonese	0.129	0.520	0.198	0.322
Response time of Cantonese	-0.114	0.570	-0.112	0.579
Auditory rhyming judgement task				
Accuracy of Mandarin	0.100	0.620	0.002	0.992
Response time of Mandarin	-0.210	0.294	-0.207	0.300
Accuracy of Cantonese	0.176	0.380	0.008	0.970
Response time of Cantonese	-0.027	0.893	-0.051	0.801
Rapid naming				
Mandarin-color	-0.079	0.694	0.165	0.410
Mandarin-digit	0.202	0.312	0.107	0.595
Mandarin-letter	0.033	0.868	-0.187	0.351
Mandarin-object	0.024	0.906	0.190	0.343
Cantonese-color	-0.214	0.283	-0.166	0.407
Cantonese-digit	0.023	0.908	-0.043	0.832
Cantonese-object	-0.069	0.731	0.157	0.434
Stroop	-0.491	**0.009****	-0.469	**0.014***
Go/nogo	-0.255	0.200	-0.176	0.379
Shift	-0.164	0.413	-0.058	0.774
Self-rated proficiency				
Cantonese-Speaking	-0.077	0.685	0.075	0.692
Cantonese-Writing	-0.288	0.123	-0.401	0.028
Cantonese-Understading	-0.070	0.711	-0.044	0.816
Cantonese-Reading	-0.119	0.530	-0.298	0.109
Mandarin-Speaking	0.092	0.628	-0.263	0.160
Mandarin-Writing	-0.041	0.829	-0.371	0.044
Mandarin-Understading	-0.178	0.346	-0.434	0.017
Mandarin-Reading	0.002	0.990	-0.294	0.115

'BG': BG > MG subnetwork (the subnetwork strongly connected in the bilinguals); 'MG': MG > BG subnetwork (the subnetwork strongly connected in the monolinguals). *p< 0.05, **p< 0.01.

Table S4. The correlation between the brain network connectivity and behavioral measures in monolinguals

	the sum strength of functional connectivity in 'BG' network		the sum strength of functional connectivity in 'MG' network	
	r	*p*	*r*	*p*
Visual rhyming judgement task				
Accuracy of Mandarin	-0.360	0.051	-0.340	0.066
Response time of Mandarin	-0.161	0.395	-0.024	0.900
Auditory rhyming judgement task				
Accuracy of Mandarin	-0.378	**0.040***	-0.418	**0.022***
Response time of Mandarin	-0.032	0.865	0.179	0.345
Rapid naming				

Mandarin-color	-0.067	0.727	-0.144	0.447
Mandarin-digit	-0.038	0.842	-0.075	0.693
Mandarin-letter	-0.140	0.461	-0.187	0.324
Mandarin-object	-0.103	0.589	0.007	0.972
Stroop	-0.046	0.826	0.159	0.447
Go/nogo	-0.097	0.644	-0.276	0.181
Shift	-0.062	0.769	-0.157	0.452
Self-rated proficiency				
Mandarin-Speaking	0.163	0.389	-0.047	0.806
Mandarin-Writing	0.138	0.466	0.007	0.972
Mandarin-Understading	0.105	0.582	0.028	0.885
Mandarin-Reading	0.278	0.136	0.274	0.143

'BG': BG > MG subnetwork (the subnetwork strongly connected in the bilinguals); 'MG': MG > BG subnetwork (the subnetwork strongly connected in the monolinguals). *p< 0.05, **p< 0.01.

References

[1] Tzourio-Mazoyer N, Landeau B, Papathanassiou D *et al.* Automated anatomical labeling of activations in SPM using a macroscopic anatomical parcellation of the MNI MRI single-subject brain Neuroimage 2002, 15:273-289. doi:10.1006/nimg.2001.0978.

[2] Rubinov M, Sporns O. Complex network measures of brain connectivity: Uses and interpretations Neuroimage 2010, 52:1059-1069. doi:10.1016/j.neuroimage.2009.10.003.

[3] Watts DJ, Strogatz SH. Collective dynamics of 'small-world' networks Nature 1998, 393:440-442. doi:10.1038/30918.

[4] Latora V, Marchiori M. Efficient behavior of small-world networks Physical Review Letters 2001, 87. doi:10.1103/PhysRevLett.87.198701.

[5] Sporns O, Honey CJ. Small worlds inside big brains Proceedings of the National Academy of Sciences of the United States of America 2006, 103:19219-19220. doi:10.1073/pnas.0609523103.

[6] Humphries MD, Gurney K. Network 'small-world-ness': a quantitative method for determining canonical network equivalence PloS one 2008, 3:e0002051. doi:10.1371/journal.pone.0002051.

[7] Ma J, Wu Y, Sun T *et al.* Neural substrates of bilingual processing in a logographic writing system: An fMRI study in Chinese Cantonese-Mandarin bilinguals Brain Res 2020, 1738:146794. doi:10.1016/j.brainres.2020.146794.

[8] Wu Y, Ma J, Cai L *et al.* Brain Activity during Visual and Auditory Word Rhyming Tasks in Cantonese-Mandarin-English Trilinguals Brain Sci 2020, 10. doi:10.3390/brainsci10120936.

[9] MacLeod CM. Half a century of research on the Stroop effect: an integrative review Psychological bulletin 1991, 109:163-203.

[10] Scarpina F, Tagini S. The Stroop Color and Word Test Front Psychol 2017, 8:557. doi:10.3389/fpsyg.2017.00557.

[11] Verbruggen F, Logan GD. Automatic and controlled response inhibition: associative learning in the go/no-go and stop-signal paradigms J Exp Psychol Gen 2008, 137:649-672. doi:10.1037/a0013170.
